# An antigen microarray protocol for COVID-19 serological analysis

**DOI:** 10.1016/j.xpro.2021.100815

**Published:** 2021-08-26

**Authors:** Joseph Longworth, Gunnar Dittmar

**Affiliations:** 1 Luxembourg Institute of Health, RPPA Platform, 1A-B, rue Thomas Edison, 1445 Strassen, Luxembourg; 2 Proteomics of Cellular Signaling, Luxembourg of Health, 1A, Rue Thomas Edison, 1445 Strassen, Luxembourg; 3 Department of Life Sciences and Medicine, University of Luxembourg, 4367 Belvaux, Luxembourg

**Keywords:** Antibody, Bioinformatics, Health Sciences, High Throughput Screening, Immunology, Proteomics

## Abstract

The emergence of the coronavirus disease 2019 pandemic increased the interest in analysis of immunoglobulin responses. ELISA and lateral flow assays are widely used but are restricted by a single response value to an antigen or antigen pool. Here, we describe antigen microarrays, an alternative allowing simultaneous assessment of multiple interactions between antigens and the immunoglobulin content of patient sera. The technique requires minimal reagents and sample input and can be adapted to a wide variety of potential antigenic targets of interest.

## Highlights

• Quantification of COVID-19 immunoglobulin response
• High-throughput analysis of patient cohorts
• Multiplexed analysis for IgG, IgA, and IgM
• R shiny data analysis software with web-based interface

## Graphical abstract

**Figure:**
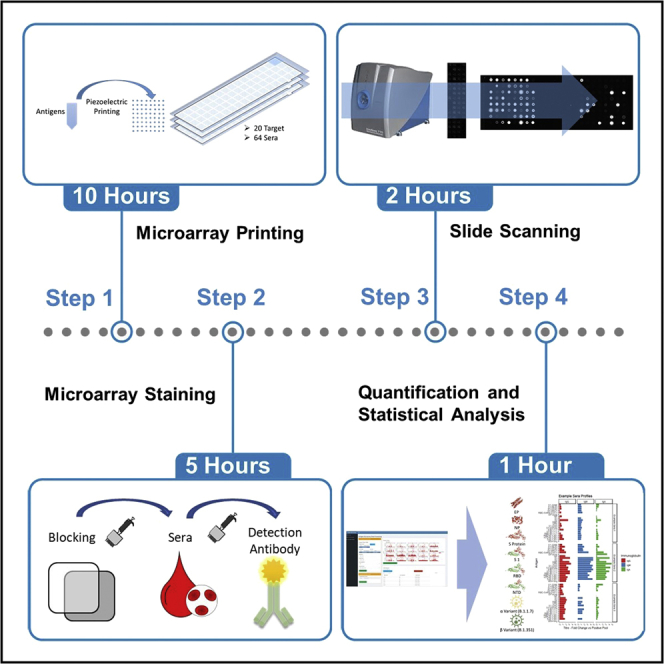

## Before you begin

The protocol below describes the steps for the production and analysis of antigen microarrays (de Assis et al., 2021; Okba et al., 2019; Reusken et al., 2013). This includes printing of arrays, staining, measuring and finally analyzing the recorded data.

First, the microarrays are printed in a 64-array format on a nitrocellulose backed microscope slide using noncontact printing on a Nanoplotter 2.1. Each array consisting of 64 spots representing a panel of 20 SARS-CoV-2 antigens (key resources table, Figure 2). Each antigen being spotted three times on the array at three dilution points. The selected proteins are the envelope protein, nucleocapsid protein and the spike protein. The spike protein (S) is included in its intact form and its fragments: S1, S2, N-terminal domain (NTD) and receptor-binding domain (RBD). Four S1 proteins from the other human coronaviruses 229E, NL63, HKU1 and OC43 (Burrell et al., 2017) were included on the array as well as the RBD of SARS-CoV-1.

To measure the response to Covid-19 variants, several recombinant protein fragments of the S protein containing mutations of B1.1.7 (Alpha variant)(S, S1 and RBD) and B.1.351 (Beta variant) (S, RBD and NTD) were included as antigen targets.

The described 20 antigens used within this study were selected as of interest and commercially available at time of production. The same procedure is however widely applicable and alternative antigens in relation to SARS-CoV-2 such as the membrane protein, Papain-like Protease or Nonstructural proteins 1, 7, 8, 10 and 16 could be included. Beyond SARS-CoV-2 research, antigen panels could include targets from other viruses, bacteria or suspected allogenic antigens.

Second, we describe the procedure for assessing a panel of 48 serum samples per slide with inclusion of controls and the detection of IgG, IgM and IgA binding. This is followed by a protocol for scanning the signals on an InnoScan 710 IR. Finally, a bioinformatics Web application toolkit written in R-based Shiny is provided and described for processing of antigen microarrays, fitting both antigen dilution series and multiple gain scans to a unified response intensity.

The positive and negative patient pools were created from serum collected in the Predi-COVID cohort with ethical approval (CNER 2022009/01).

### Prepare buffers and reagents

**Timing: 0.5–4 h**

1. If needed, prepare buffers following the recipes in the “materials and equipment”. Make sure that there is enough of all needed solutions, apart from those indicated as “prepare fresh on the day”.
2. Prepare 50 μL aliquots of the antigens and store at −80°C until ready for use (up 12 months). Here we describe the preparation of the purchased recombinant protein fragments stocks.
  a. Reconstitute lyophilized proteins a final concentration of 0.25 μg/μL using ddH_2_O.
  b. Incubate on a rocker for 30 min until fully dissolved.

***Note:*** Antigens, which are not fully dissolved, will disturb the printing process.

3. Prepare 50 μL aliquots of immunoglobulin (IgG, IgM and IgA) controls and store at −80°C until ready for use (up to 12 months).
  a. Reconstitute Immunoglobulin G, M and A to a final concentration of 0.75 mg/mL in a 50:50 mix of ddH_2_O: glycerol.
  b. Aliquot immunoglobulin solutions into 50 μL and store at −80°C until ready for use.

***Note:*** The inclusion of three immunoglobulins as well as a mixed pool as controls at the corner of each array both aids in assessment of secondary staining but also aids in image recognition during quantification of the arrays.

***Note:*** The protocol below describes the use of 20 specific antigens of interest to the research of Covid-19. If a different list of target antigens is to be investigated, optimization of the printing concentration may be necessary.

## Key resources table

**Table:** 

REAGENT or RESOURCE	SOURCE	IDENTIFIER
**Antibodies**		

Goat Anti-Human Serum IgA Alexa Fluor 790, working concentration: 0.75mg/mL	Jackson ImmunoResearch Laboratories	Cat#109-655-011; RRID:AB_2889014
Goat Anti-Human Serum IgG Alexa Fluor 790, working concentration: 0.75mg/mL	Jackson ImmunoResearch Laboratories	Cat#109-655-190; RRID:AB_2889015
Goat Anti-Human Serum IgM Alexa Fluor 790, working concentration: 0.75mg/mL	Jackson ImmunoResearch Laboratories	Cat#109-655-129; RRID:AB_2337908
Anti-SARS-CoV-2 RBD Potent Neutralizing Antibody, Human IgG1 (AM18), working concentration: 0.75mg/mL	ACROBiosystems	Cat#SPD-M180

**Chemicals, peptides, and recombinant proteins**		

SARS-CoV-2 S protein RBD, His Tag (MALS verified)	ACROBiosystems	Cat#SPD-C52H3
SARS-CoV-2 S1 protein NTD, His Tag	ACROBiosystems	Cat#S1D-C52H6
SARS-CoV-2 Envelope protein, GST,His Tag	ACROBiosystems	Cat#ENN-C5128
SARS-CoV-2 Nucleocapsid protein, His Tag	ACROBiosystems	Cat#NUN-C5227
SARS S protein RBD, His Tag (MALS verified)	ACROBiosystems	Cat#SPD-S52H6
SARS-CoV-2 Spike S1(D614G)-His Recombinant Protein, HPLC-verified	Sino Biological	Cat#40591-V08H3
Human coronavirus HKU1 (isolate N1) (HCoV-HKU1) Spike/S1 Protein (S1 Subunit, His Tag)	Sino Biological	Cat#40021-V08H
Human coronavirus (HCoV-229E) Spike/S1 Protein (S1 Subunit, His Tag)	Sino Biological	Cat#40601-V08H
Human coronavirus (HCoV-NL63) Spike/S1 Protein (S1 Subunit, His Tag)	Sino Biological	Cat#40600-V08H
Human coronavirus (HCoV-OC43) Spike S1 Protein (His Tag)	Sino Biological	Cat#40607-V08H1
SARS-CoV-2 S1 protein (HV69-70del, N501Y, D614G), His Tag	ACROBiosystems	Cat#S1N-C52Hk
SARS-CoV-2 S protein RBD (N501Y), His Tag (MALS verified)	ACROBiosystems	Cat#SPD-C52Hn
SARS-CoV-2 S protein (HV69-70del, Y144del, N501Y, A570D, D614G, P681H, T716I, S982A, D1118H), His Tag (MALS verified)	ACROBiosystems	Cat#SPN-C52H6
SARS-CoV-2 S1 protein NTD (L18F, D80A, D215G, R246I), His Tag	ACROBiosystems	Cat#S1D-C52H9
SARS-CoV-2 S protein (L18F, D80A, D215G, R246I, K417N, E484K, N501Y, D614G, A701V), His Tag (MALS verified)	ACROBiosystems	Cat#SPN-C52Hc
SARS-CoV-2 S protein RBD (K417N, E484K, N501Y), His Tag (MALS verified)	ACROBiosystems	Cat#SPD-C52Hp
SARS-CoV-2 Spike S1-His Recombinant Protein	Sino Biological	Cat#40591-V08B1
SARS-CoV-2 S2 protein, His Tag	ACROBiosystems	Cat#S2N-C52H5
S1-CHO	(Johari et al., 2021)	N/A
Tween™ 20	Fisher Scientific	Cat#BP337
FBS	Thermo Fisher Scientific	Cat#10500-064
Glycerol	Fisher Scientific	Cat#BP229-1
Dimethyl sulfoxide	Fisher Scientific	Cat#10213810
TBS, Tris Buffered Saline, 10× Solution, pH 7.4	Fisher Scientific	Cat#BP24711

**Software and algorithms**		

R	R Project	RRID:SCR_001905 version #4.0.3
Mapix	Innopsys	RRID:SCR_002723 version #9.0.0
NP2.1 S/N:2181	GeSiM	N/A

**Deposited data**		

Code and example datasets	This study	https://github.com/JosephLongworth/AntigenMicroarray

**Biological samples**		

Positive patient pool	Predi-COVID study.(Fagherazzi et al., 2020)	N/A
Negative patient pool	Predi-COVID study.(Fagherazzi et al., 2020)	N/A

**Other**		

384 Square well plate	Greiner	Cat#781201
Aluminium StarSeal (PCR)	STARLAB	Cat#E2796-9792
Nano-Plotter^TM^ 2.1	GeSiM	N/A
Chiller	GeSiM	Cat#Ministat125
Humidifier	GeSiM	Cat#AirwinC11
ONCYTE® SuperNOVA 64 Pads	Grace Bio-Labs	Cat#705064
Normal Human Serum Control	Fisher Scientific	Cat#10406144
384 Well Deep Well Plate	Corning	Cat#3342
quadriPERM® 4 - slide plate	SARSTEDT	Cat#94.6077.307
ProPlate® multi-well chambers 64 well slide	Grace Bio-Labs	Cat#248865
ProPlate® Multi-Array Slide System	Grace Bio-Labs	Cat#249865
InnoScan 710 Microarray Scanner	Innopsys	Cat#InnoScan 710-IR

## Materials and equipment

**Table:** 2× Print Buffer

Reagent	Final concentration	Amount
Glycerol	40%	200 mL
DMSO	2M	71 mL
ddH_2_O	n/a	229 mL
**Total**	**n/a**	**500 mL**

Store at 4°C (maximum 3 months).

**Table:** TBS

Reagent	Final concentration	Amount
10× TBS	1×	50 mL
ddH_2_O	n/a	450 mL
**Total**	**n/a**	**500 mL**

Store at 19°C–22°C (maximum 3 months).

**Table:** TBS-T

Reagent	Final concentration	Amount
10× TBS	1	50 mL
Tween 20	0.1%	500 μL
ddH_2_O	n/a	449.5 mL
**Total**	**n/a**	**50 mL**

Store at 19°C–22°C (maximum 3 months).

**Table:** TBS-T 10%FBS

Reagent	Final concentration	Amount
TBS-T	n/a	45 mL
Tween 20	10%	5 mL
**Total**	**n/a**	**50 mL**

Prepare fresh on the day of the experiment.

**Table:** Print Buffer 10% FBS

Reagent	Final concentration	Amount
2× Print Buffer	1×	25 mL
FBS	10%	5 mL
ddH_2_O	n/a	20 mL
**Total**	**n/a**	**50 mL**

Prepare fresh on the day of the experiment.

**Table:** TBS-T 10%FBS

Reagent	Final concentration	Amount
2× Print Buffer	n/a	45 mL
Tween 20	10%	5 mL
ddH_2_O	n/a	450 mL
**Total**	**n/a**	**500 mL**

Prepare fresh on the day of the experiment.

## Step-by-step method details

### Microarray printing

**Timing: 10 h**

**Figure 1:**
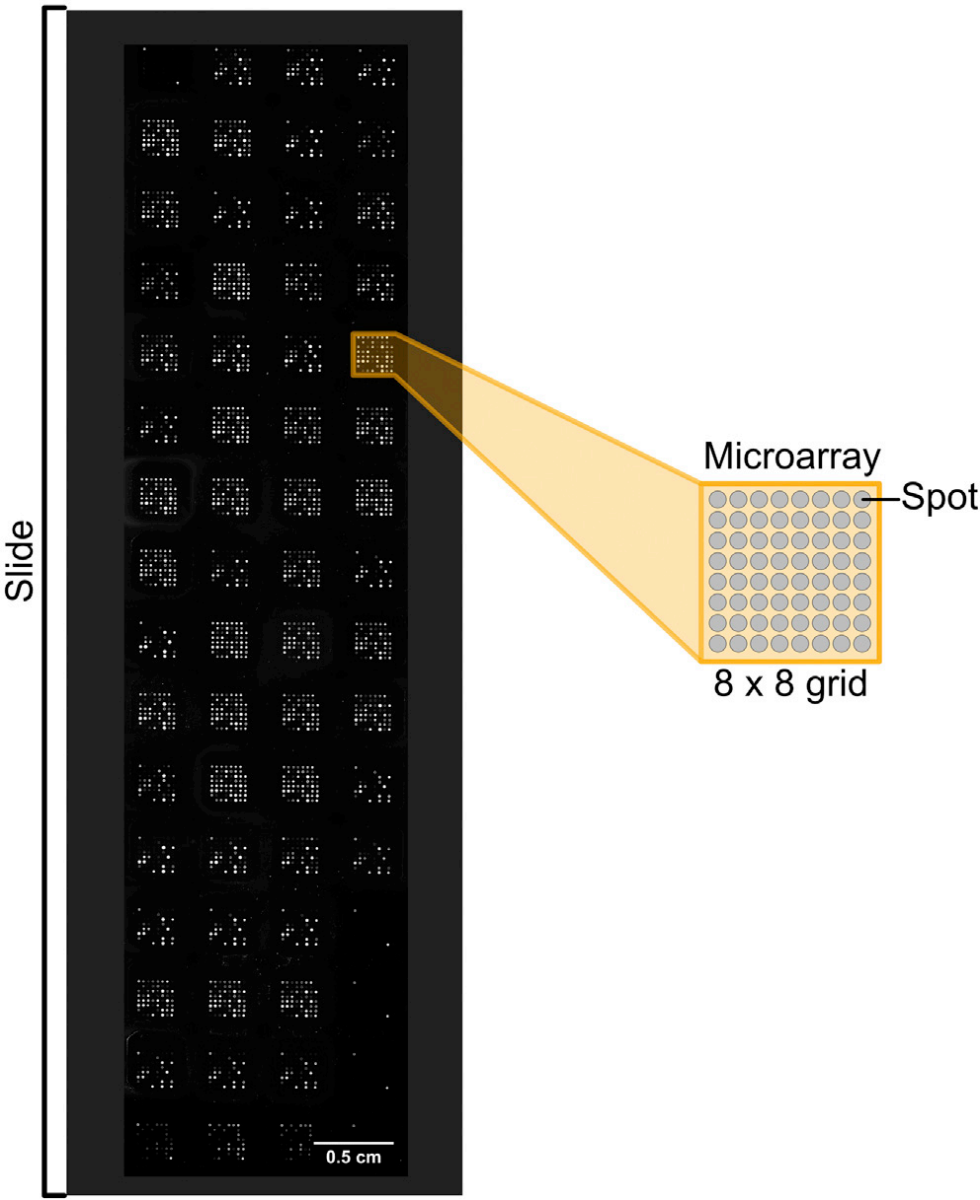
Example of a 64 array with 8 × 8 matrix

***Alternatives:*** This protocol describes the use of an antigen microarray incorporating 64 microarray slides printed with microarrays of 8 by 8 spots (Figure 1). Alternative formats can be used such as 24, or 16 and 8 microarray slides allowing for the increase in targets included on each microarray though requiring the reduction of samples assessed per microarray slide. The provided bioinformatic application can be adjusted to facilitate the design of such arrays.

***Alternatives:*** This protocol describes the utilization of a GeSim Nanoplotter 2.1 for the production of microarrays. Other contactless and contact printers are available and can be utilized as an alternative following the operation instructions of the Nanoplotter available.

In this step, the user will prepare the antigen microarrays. This includes setting up the GeSim Nanoplotter, running a ‘warm-up’ test print and preparing of a ‘Print Plate’ with the antigens of interest.

1. Set up the GeSim Nanoplotter 2.1. for printing.
  a. Preparing the GeSim Nanoplotter 2.1 for printing.
    i. Turn on the nanoplotter.
    ii. Turn on the chiller to cool the printing surface to 17°C.
    iii. Turn on the humidifier to 60% relative humidity.
    iv. Wait for 1 h for the print environment to stabilize.
    v. Switch on the computer and launch the NP2.1 software.
    vi. Under “System” select “Initialize Nano-Plotter” allowing it to run through its start-up processes.
    vii. Whilst in “Interactive mode” under “Work Plate” select “Open Work Plate settings” and load a work plate file ∗.npw (Data S1) describing the physical layout of the nanoplotter with slides detailing 64 microarrays per slide with a 8 by 8 grid layout matching the dimensions of the 64 microarray ONCYTE® SuperNOVA.
  b. Place glass slides in the nanoplotter for a ‘warm-up’ print run.
    i. Fill column A of a 384 square well plate with 1× Print Buffer to create a print plate.
    ii. Place the prepared print plate on the nanoplotter and select as “usable” by right clicking the corresponding location in the GeSIM software.
    iii. Place one (or more) blank glass slide onto the slide deck of the nanoplotter and select as “usable” by right clicking the corresponding location in the GeSIM software.
  c. Run a ‘warm-up’ print run.
    i. Make sure the software is in “Run” mode.
    ii. Under “Program” select “Run NPL-Application”.
    iii. Select a “TransferSimMultiPlates_06A1” program with default parameters and the ‘Print Plan’ provided as Data S2 or prepared using the provided R-based Shiny Software provided.

***Note:*** A visual inspection of the printed slides can be done by eye to ensure correct printing by holding the slide to the light. Printed arrays should form an aligned grid of droplets without missing positions. If the piezoelectric tip has not been in use for some time, the tip is dirty or has degraded, then droplets may not emit from the piezoelectric tip reproducibly or may emit with an unacceptable diffraction. Such issues are commonly observed after a period of inactivity of a few day but are resolved after 2–3 blank print runs.

**Figure 2:**
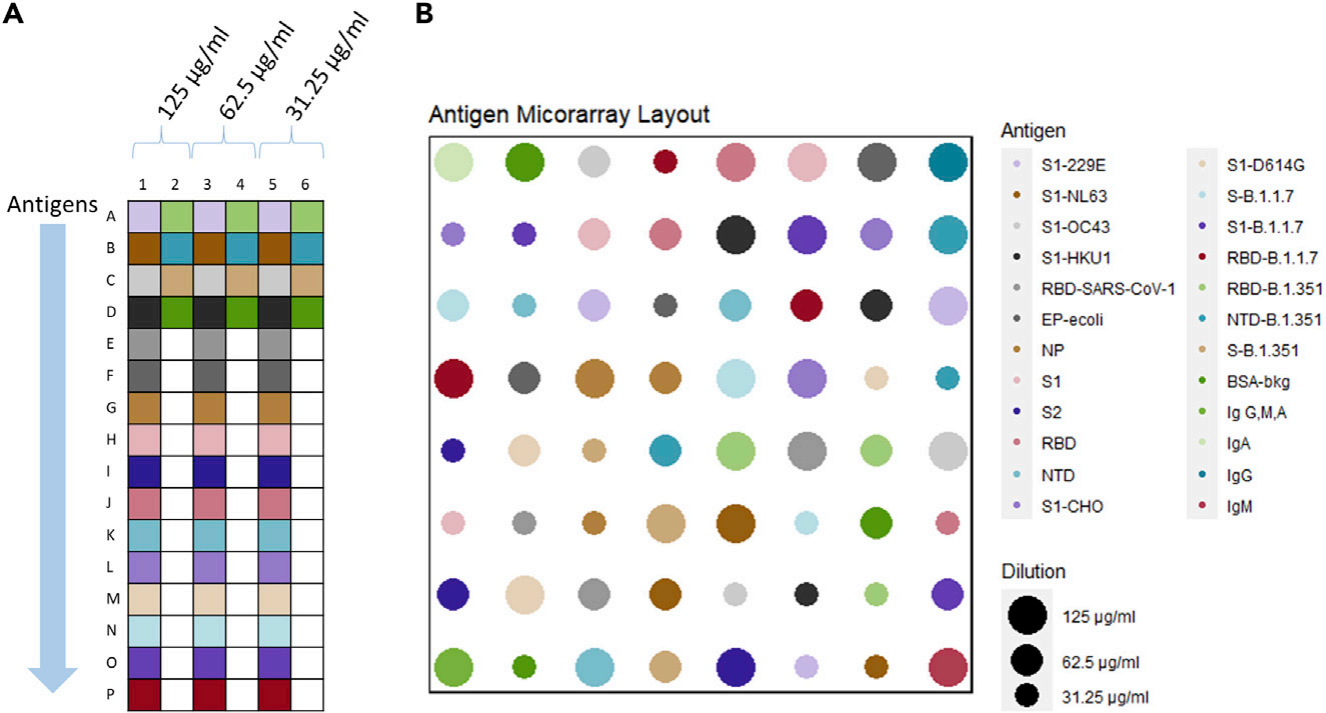
Schematic of print plate layout along with corresponding array layout using the provided print plan and print design (A) Layout of the print plate. 20 antigens and their dilutions are placed in alternating positions as indicated by the different colors. (B) Arrangement of the antigens on the microarray. Antigen from the print plate has the same color in the microarray arrangement. The 20 antigens included can be seen in different colors, each represented by three spots. The different sizes are representing different dilutions. Layout of the array is randomized to prevent spatial effects. If a new print plan and print design was produced by the provided shiny-based application, the layout would be altered correspondingly.

2. Prepare a ‘Print Plate’ from which microarrays will be printed (Figure 2).
  a. Prepare a solution of 1× Print Buffer by diluting 10 mL of 2× Print Buffer with 10 mL ddH_2_O and add to a pipette basin.
  b. Using a 16-channel pipette dispense 50 μL of 1× Print Buffer to columns 3 to 6 of a 384 square well plate.
    ***Note:*** These columns contain the diluted antigens and location is based on the print plan assuming a dilution shift of 32.
  c. Dilute each antigen with 50 μL of 2× print buffer before transferring sequentially down column 1 and 7 of a 384 square well plate.
  d. Using a 16-channel pipette prepare a serial dilution of the antigen by aspirating 25 μL from column 1 and mix in column 3. Mix by pipetting 50% of the solution 5 times in the same tip avoiding foam creation.
  e. Repeat this procedure from column 2 to 4. Then using fresh tips from 3 to 5 and 4 to 6.

3. Add Immunoglobulin controls to the print plate.
  a. Dilute immunoglobulins G, M and A with 50 μL print buffer and add to wells A24, B24 and C24 respectively.
  b. Transfer 25 μL of immunoglobulins added to the print plate above and mix into well D24.

**Pause point:** Once the print plate is prepared, it can be sealed using an adhesive Aluminium StarSeal attached to the top of the 384 square well plate and stored at −20°C for later use or after a print run for re-use in a subsequent print run.

4. Prepare ‘Print Plan’.
  a. To print the microarrays using a GeSim Nanoplotter a print plan must be created describing the arrangement of spots for the microarray printer to print. An example file is included in Data S3.

***Optional:*** Different arrangements of the antigens can be created using the R-based Shiny tool provided.

5. Load the nanoplotter.
  a. If the print plate was frozen before the print, place the plate on ice to thaw for 15 min on ice.
  b. Centrifuge the print plate at 100 x g for 5 min at room temperature (18°C–24°C).
  c. Remove the aluminium cover placing on the print plate of the Nanoplotter and selecting as “usable” in the GeSIM software.
  d. Place as many ONCYTE® SuperNOVA 64 pad slides as desired for the print run onto the Nanoplotter. Push each slide to the top right corner of the stops so each slide consistently aligns and select as “usable” in the GeSIM software.
  e. Run the nanoplotter.
    i. Whilst in “Run” mode under “Program” select “Run NPL-Application”.
    ii. Select a “TransferSimMultiPlates_06A1” program with default parameters and the ‘Print Plan’ that was prepared above.
6. Removal of printed microarrays.
  a. Remove printed microarrays and store at 4°C.

***Note:*** Printed microarrays should be left for at least 12 h at 4°C until the spots are fully absorbed by the nitrocellulose surface.

**Pause point:** The printed microarrays can be stored at 4°C for up to a month. Do not freeze!

### Microarray staining

**Timing: 5 h**

This section describes the preparation of the serum samples for the analysis. The antigen microarrays are blocked before incubation with the patient sera. Following the incubation step, the microarrays are washed. Finally, the bound antibodies are detected with a fluorescently labeled secondary antibody and read on a scanner.

7. Prepare a control mixture.
  a. Pool together a representative pool of positive patient sera for the cohort being investigated to create a positive pool control for the cohort. The positive control pool will serve as the positive reference. Create 100 μL positive control pool aliquots and store them at −20°C for up to one year or longer at −80°C.
  b. Pool together multiple negative patient sera to derive a negative pool control for the cohort. The negative control pool will serve as the negative reference. Create 100 μL negative control pool aliquots and store them at −20°C.

***Note:*** The experimenter has to ensure that the positive and the negative pool are representing valid controls for the experiments. In the presented example the positive sera pool was collected from patients who were tested positive in a Covid-19 PCR test. The negative pool was assembled at an early stage of the COVID-19 pandemic, where exposure to Covid-19 can be assumed to be low.

The microarray contains additionally four other human Corona virae (HCoV). For these samples the negative pool will generate still signals as virus infections with HCoVs are common. For these samples a proper negative control would be the “no serum” control.

***Optional:*** Reconstitute pre-2019 normalized human serum and dispense into 50 μL aliquots.

**Figure 3:**
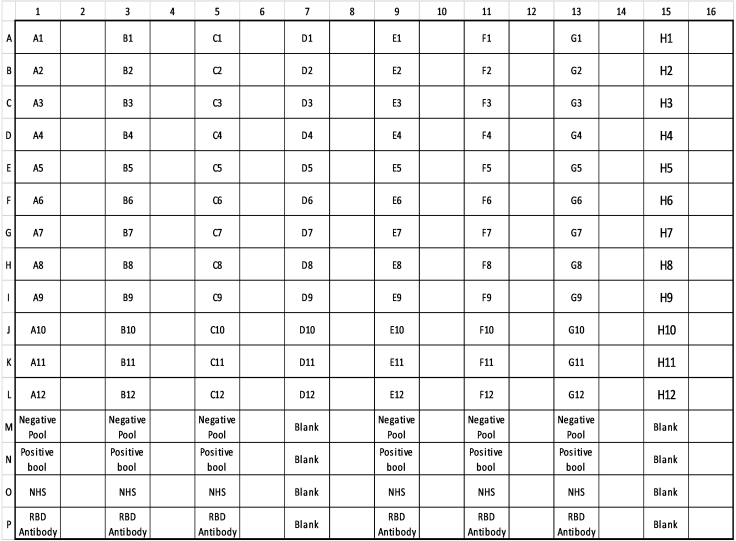
Schema of the layout of a sample plate Designed to accommodate 96 serum samples and to be processed on two 64 array antigen microarrays.

**Figure 4:**
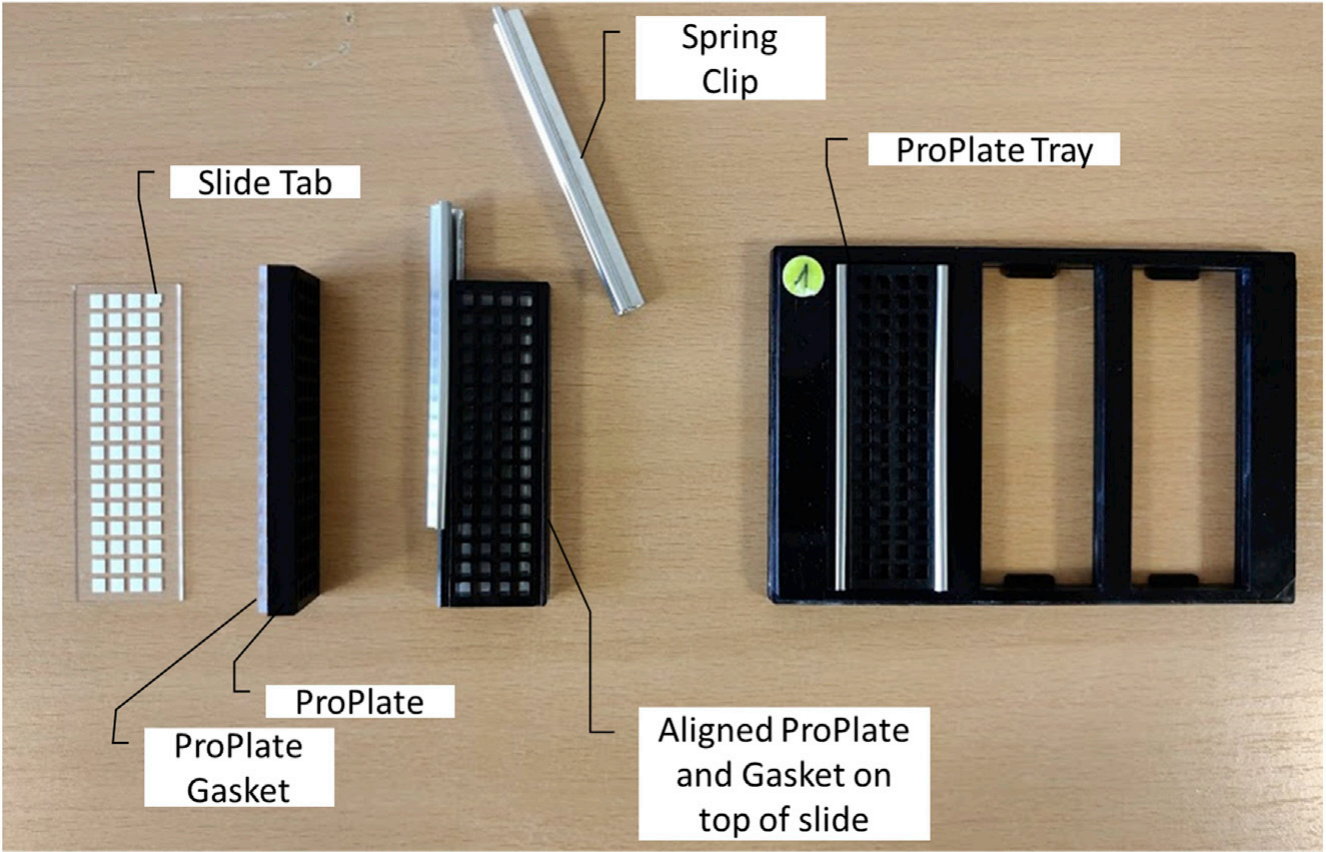
Image of the antigen microarray slide, ProPlate® multi-well chambers 64 well slide, spring clips and ProPlate® multi-array slide system Clipping the ProPlate on the Antigen microarray slide creates wells, which can be used for the incubation with different sera.

8. Prepare a sample plate of serum samples (Figure 3)
  ***Note:*** The antigen microarrays slides have 4 columns of 16 arrays allowing 64 sera assessments per slide. Reserving 16 arrays per slide for controls, a collection of 48 serum samples can be assessed per slide. Figure 3 shows an example layout optimized for the transfer of half of a 96 well sample plate/box to form a sample plate.
  a. Using a 16-channel pipette, add 190 μL of TBS-T with 10% FBS to the first 8 odd-numbered columns of a 384 Well Deep Well Plate (Corning, Cat#3342).
  b. Add 10 μL of the negative pool to wells of row M previously loaded with buffer mixing by pipetting up and down gently 5 times.
  c. Add 10 μL of the positive pool to wells of row N previously loaded with buffer mixing by pipetting up and down gently 5 times.
  d. Add 10 μL of normalized human serum to wells of row O previously loaded with buffer mixing by pipetting up and down gently 5 times.
  e. Add 10 μL of up to 96 serum sample to be assessed to rows A-L previously loaded with buffer mixing by pipetting up and down gently 5 times. If using samples stored in a 96 well/box format samples can be transferred using an 8 channel pipette rotated as shown in Figure 3.
  f. Add aluminum foil tape to the top of the plate to seal.
    **Pause point:** After preparation of the sample plate, the plate can be stored at −20°C for up to 3 months, allowing the processing on a subsequent day. If the sample plate is to be used on the same day it should be stored at 4°C to avoid freeze/thaw cycles.

9. Block antigen microarrays
  a. To measure the different immunoglobulins (IgG, IgA, IgM) of the patient sera use three antigen microarrays per Immunoglobulin. Place each into a separate chamber of a quadriPERM® 4 - slide plate.
  b. Gently add 4.5 mL TBS-T and place on a gentle rocker for 30 min.
  c. Remove TBS-T buffer.
  d. Gently add 4.5 mL Print Buffer with 10% FBS and place on a gentle rocker for 30 min.
  e. Remove Print Buffer with 10% FBS buffer.
  f. Gently add 4.5 mL TBS-T with 10% FBS and place on a gentle rocker for 60 min.

10. Attach ProPlate (Figure 4)
  **CRITICAL:** It is important that the slides are not drying out during the incubation. Use one slide at a time to ensure rapid processing (see troubleshooting point number 5 for further details).
  a. Remove the slide from the blocking plate and add a 64 well ProPlate on top of the slide aligning the nitrocellulose pads and the wells of the ProPlate. Keeping the Slide Tab to the upper right corner.
  b. Add the spring clips and ensure the wells are still well aligned with the slide pads.
  c. Place the slide with ProPlate frames into the ProPlate Tray maintaining the tab to the top right corner.
  d. Using a 16-channel pipette add 40 μL TBS-T with 10% FBS.

11. Serum incubation
  **CRITICAL:** Handling of serum must be carried out in appropriate safety conditions for primary human samples such as serum.
  a. If the sample plate was frozen prior to use, place at 4°C until thawed.
  b. For each antigen microarray slide transfer 10 μL of sample from four columns of the sample plate prepared in step 8. Add aluminum foil tape to the top of each slide to seal.
  c. Incubate slides for 3 h on a rocker at room temperature with gentle agitation.
  d. Return the sample plate to the −20°C unless the second batch of three slides is being assessed concurrently.

12. Remove Serum
  a. Using the 16-channel pipette set to 100 μL, remove the majority of fluid from each well.
  b. Use the “pipette and mix” function to add 50 μL TBS-T to each well, cycling 25 μL 3 times to mix the well-contents.
  c. Repeat steps 14a and 14b for a total of 3 times to exchange the fluid.
  d. Using the 16-channel pipette set to 100 μL remove the majority of fluid from each well.
  e. Remove the ProPlate transferring the slide into a quadriPERM® 4 - slide plate.
  f. Gently add 4.5 mL TBS-T.
  g. Exchange the buffer in the plate for fresh TBS-T. Incubate for 3 min on the rocker with gentle agitation.
  h. Repeat the previous step for a second incubation.
  i. Exchange the buffer in the plate for fresh TBS-T 10% FBS.
13. Detection antibody staining
  a. Immediately prior to use prepare the detection antibody by adding 30 μL of the selected detection antibody (IgG, IgM, IgA) to 12 mL of TBS-T 10% FBS.
  b. Gently add 4 mL of the prepared detection antibody and place on a rocker with gentle agitation for 30 min at room temperature. Cover to protect from light.
  c. Remove antibody solution from the plate and gently add 4mL of TBS-T.
  d. Remove TBS-T from the plate and gently add 4mL TBS-T. Incubate on the rocker for 3 min.
  e. Remove TBS-T from the plate and gently add 4mL TBS.
  f. Remove TBS from the plate and gently add 4 mL TBS. Incubate on the rocker for 3 min.
  g. Remove slides from quadriPERM® 4 - slide plate placing into a 50 mL tube. Gently fill with ddH_2_O close and invert gently 5 times.
  h. Remove the slide from the 50 mL tube careful not to touch the microarrays and blow-dry with a gentle stream of nitrogen.

**Pause point:** After drying, the slides may either be scanned immediately or stored in the dark at 4°C until ready to scan. Scanning up to 7 days later has a negligible effect on quantification.

### Slide scanning

**Timing: 2 h**

In this section the stained slides are scanned. Scanning is performed across several detection gains to cover a wide range of binding conditions. After scanning, the intensities are quantified through the image recognition software.

14. Prepare Scanner.
  a. Load the Mapix Software and switch on the InnoScan 710 Microarray Scanner.
  b. Connect the Mapix software to the scanner selecting “Scanner>Connect”.
  c. Gently insert a validation slide into the instrument and select “Scanner>Check Scanner” to validate the current performance of the scanner.

***Note:*** The Scanner takes some time to warm up to the working temperature. Running the validation slide 15 min before the first analysis slide is advantageous allowing the scanner’s temperature controls to normalize.

**Figure 5:**
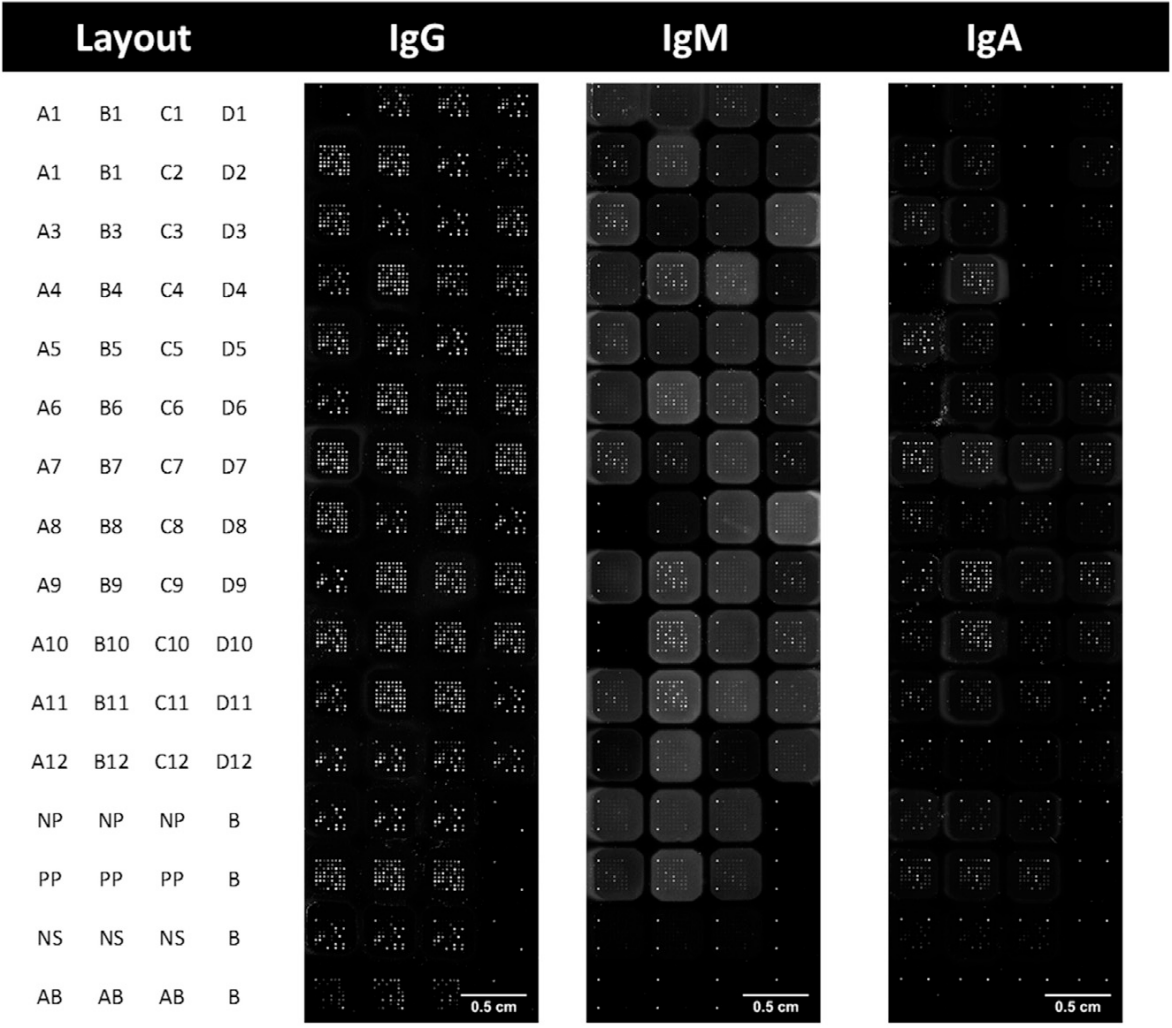
Example slides of an antigen microarray scanned at a gain of 50 Layout of serum on showing inclusion of 48 serum samples with controls NP (Negative Pool), PP (Positive Pool), NS (Normalized Serum), AB (RBD antibody) and B (Blank).

15. Prepare a batch method.
  a. Open the batch editor to begin sampling multiple gains. “Batch>Batch editor”.
  b. Open an existing method (an appropriate ∗.mbt is provided in Data S4).
  c. Alternatively, a new batch method can be created.
    ***Optional:*** Creation of a new batch method:
  d. Select Add Scan Step.
  e. Under “Scan Parameters”.
    i. Select slide layout as “Nitrocellulose 1 Pad slide”.
    ii. Click “Set scan area”.
    iii. Select pixel size as “5.0”.
    iv. Set Speed as “35”.
    v. Set Scan Mode as “Normal”.
    vi. Set Detection as “Smooth”.
    vii. Set Acquisition Mode to “785 only”.
    viii. Set the Gain as 0.
    ix. Set Laser power to “high”.
  f. Under “Filename & Directory”.
    i. Provide an appropriate filename for the scan.
    ii. Provide a folder where the scan should be saved.
    iii. Select “Export folder to all steps”.
  g. Next to step 1 on the step list click to duplicate the step. This will duplicate the parameters so only the gain needs to be changed within each step. Gains of 0,1,5,10,20,25,50,75 and 100 were used to record the data provided here.

16. Scan slides.
  a. Gently insert the analysis slide to be scanned into the scanner with a tab to the front right as orientated when loading the samples.
  b. A preview scan of the slide can be performed to visually assess the staining of the slides by selecting “Scanner>Preview whole slide”.
  c. Run the batch method by opening and selecting “Start”.
  d. Upon completion of all steps of the batch scan method, the slide can be returned to the dark at 4°C for potential rescan. An example of a scanned array is provided in Figure 5.

17. Quantify Images.
  a. Open the batch editor of Mapix.
    ***Note:*** The Scanner does not need to be on whilst processing analysis steps.
  b. Click “Add Analysis Step”.
  c. Under “Quantification”.
    i. Select an appropriate GAL file. This can be obtained from the Nanoplotter. An appropriate file for the 64 array format used here is provided as Data S5.
    ii. Under “find all automatically” select “Grid + Block + Spots”.
    iii. Under “Gridding” set max position offset (% pitch)” to 30 “Min. Diameter (% Dth)” to 50 and “Max. Diameter to (% Dth)” to 200.
    iv. Under “Photometric calculations” set “S/B border width (pixels)” to 2 and “Background calculation” to “Locally” with “Background diameter (pitch)” set to 2.
    v. Under “Save results” select “File Format” to “Mapic Results files (∗.txt)” selecting “All blocks in a file” with desired number format.
    vi. If the desired select “Save a JPEG image containing analyzed spots”.
  d. Under “Files”.
    i. Select the ∗.tif images of the scanned arrays.
  e. Run the batch method by selecting “Start”.

## Expected outcomes

To aid in understanding of the presented protocol we have included a data for nine stained slides, three each stained for response to IgG, IgM and IgA. Each slide includes 48 uknown sera samples from patients collected as part of the Predi-COVID study (Fagherazzi et al., 2020).

### Slide scans

Upon scanning of the antigen microarrays, a TIFF image file is produced. Examples of which are shown in Figure 5 for IgG, IgM and IgA. These provide initial overview of the arrays. With the print plan used in the example shown the detection antibody used is identifiable by the binging of the control corner spots. Due to the nature of the antigens selected and the patients tested that the response to IgG will be stronger than that of IgM or IgA is expected for most samples. This is particularly true for spots associated with the HCoV antigens 229E, HKU1, OC43 and NL63. Further, these will be the spots observed within arrays threated with the negative pool (NP) and normalized serum (NS).

### Data processing

**Figure 6:**
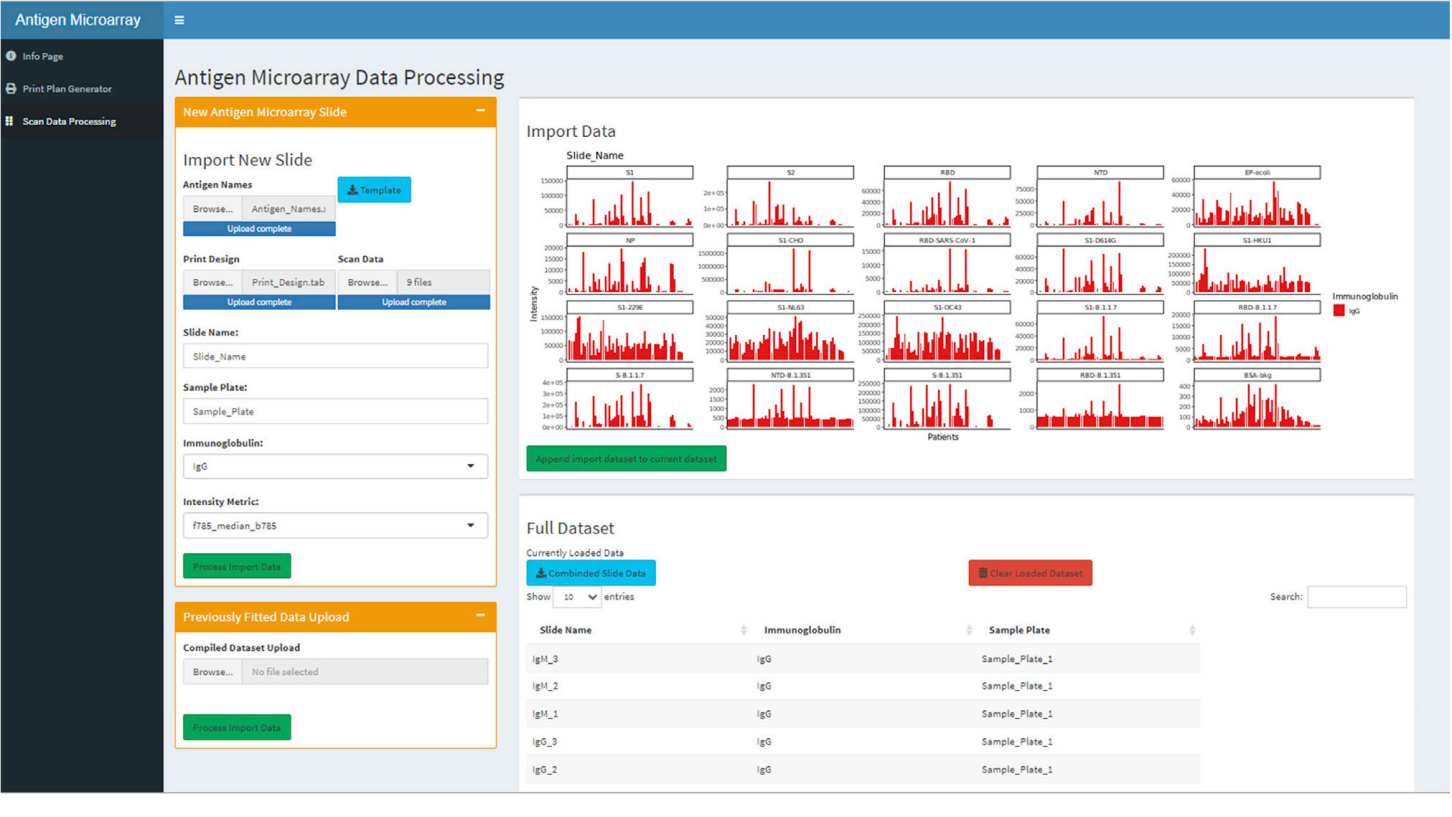
Screenshot of the antigen microarrays shiny-based graphical user interphase

In the methodology presented, we utilize multiple dilution points for the antigens and scan across a series of gains. This is done to extend the dynamic range of the assay. This is of particular importance to cover different immunoglobulin and antigens within the same assay, although it complicates the data processing. We have provided a shiny based application for processing of such data to a unified value. Figure 6 show a screenshot of the application in operation with a snapshot of the uploaded data observable in the imported data box.

### Biological interpretation

Although microarray-based assay can provide absolute quantifications, it would require the antibody validation for each antigen with known concentrations. Here we are focusing on relative quantification of the antibodies between samples and normalization to the included controls.

For each slide a threshold was determined for the background binding. For SARS-CoV-2 antigens this was set at two standard deviations above the maximal observed for the negative pool. For the HCoV antigens where the negative pools samples cannot be considered negative, the background threshold was set as two standard deviations above the maximal observed for the untreated arrays. All signals below threshold were set to non-detected.

Included on all slides in the cohort is a positive pool derived from the cohort and assessed in triplicate. This pooled sample is used for normalization of the entire cohort in order to provide comparable values across the cohort at a similar scale for each antigen and immunoglobulin. Alternative approaches could be normalizing by the native pool samples particularly where positive pool sample consistency throughout the cohort is in doubt. In such a case use of z scoring or other normalization method is suggested to align response values between antigens and immunoglobulins.

**Figure 7:**
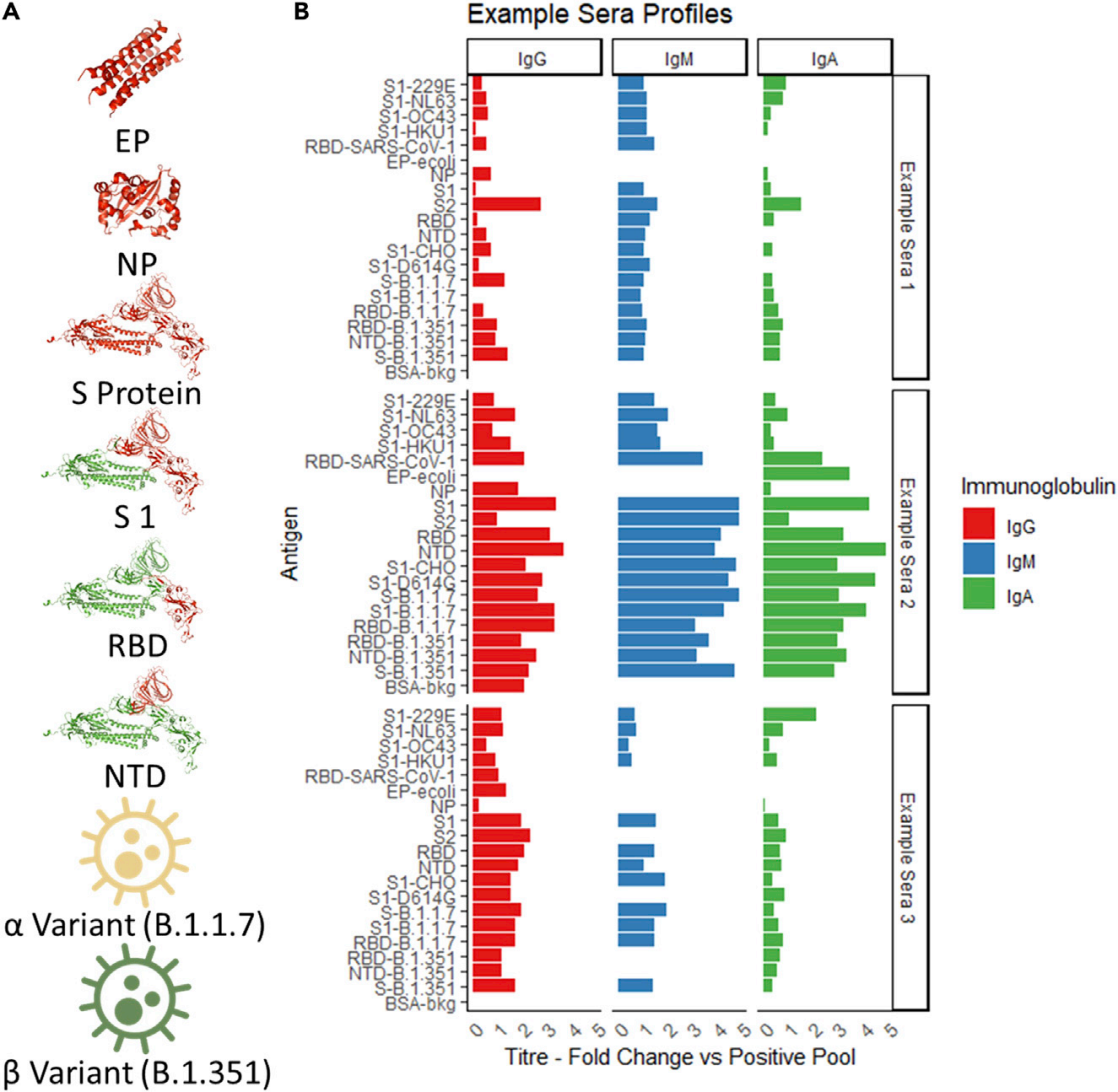
Antigens included in the microarray and quantification examples (A) Collection of antigens included within the antigen array including Envelope Protein (EP) (Mandala et al., 2020), Nucleocapsid Protein (EP) (Jayaram et al., 2006) and Spike Protein (S) (Benton et al., 2021) with fragments of the Receptor binding domain and N-Terminal domain included as separated antigen targets on the array shown in red. (B) An overview of 3 patient profiles showing response profiles to all the included antigens for each immunoglobulin.

**Figure 8:**
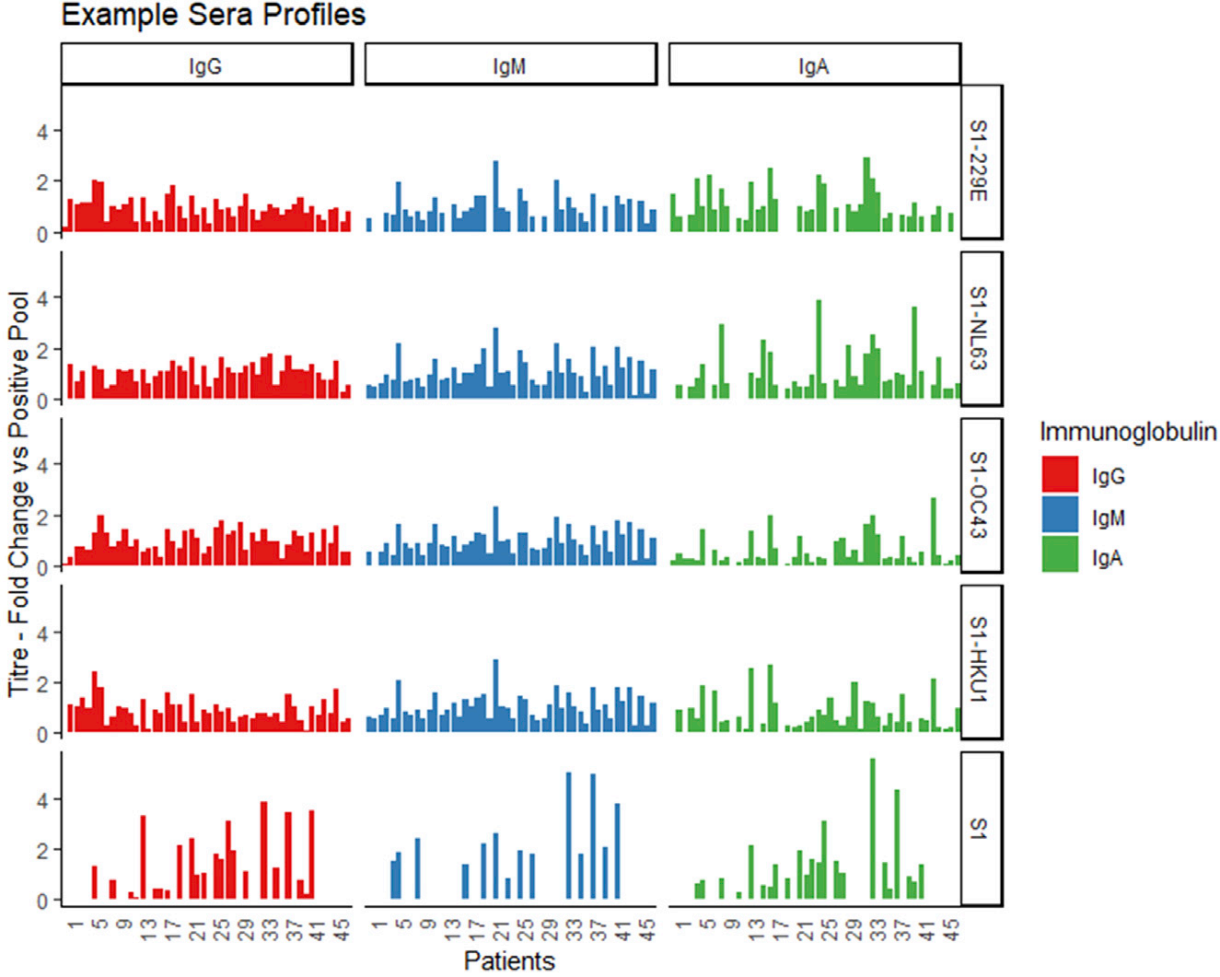
An overview of the observed response for 48 patient sera to the S1 fragment of spike protein for each of the four HCoV viruses as well as SARS-CoV-2

Analysis from the large dataset of an antigen microarray can be considered in two modes. A patient centered mode, observing the profile of antigen recognition within each sample (Figure 7). In the example of three patients, shown differences can be observed in the total recognition of antigens with example sera 2 having a stronger response than example sera 1 and 3. Alternatively, an antigen centered analysis can be performed observing the changes across a cohort in recognition of several antigens as is shown in Figure 8.

## Quantification and statistical analysis

**Timing: 1 h**

The scanned data can be further processed using the tabulated data. The scanned results are varying according to the antibody titer of the serum sample. To fit and normalize the data we recommend the in-house developed R-based Shiny application with graphical user interphase available at https://github.com/JosephLongworth/AntigenMicroarray. To aid understanding of the tool we added example files in the supplemental information (see Table 1 below). The software can process the spot intensity data table reported from the Mapix and other scanner software using the Genpix format. Scan data is first associated with descriptive data of the slide design used during the printing procedure as well as description of the antigen targets printed. The R-package SuperCurve (Coombes et al., 2017) used for RPPA (reverse phase protein array) analysis is then utilized to fit a logistic regression model against the antigen dilution series for each antigen and gain across the full dataset. This fit of the full dataset is known as a supercurve. Each sample antigen dilution series is fitted to the derived supercurve, thus determining a single intensity for each sample, antigen and gain combination. A linear model is then fitted to the intensities observed over different gains, removing saturated values. Based on this curve an intensity observed for each antigen and serum samples at the selected gain(default set to 50) a gain of 50 is reported back as the gain normalized intensity.

1. Install the shiny application
  a. Following the instruction on Github (https://github.com/JosephLongworth/AntigenMicroarray), download install and launch the shiny-based web GUI within a shiny server or as a standalone process on an individual computer.

2. Upload a new antigen array slide data and fitting the data to derive single antigen serum binned intensity.
  a. Navigate to the Scan Data Processing section in the taskbar on the left side.
  b. Select “New Antigen Microarray slide” to open the menu.
  c. Upload an antigen names table describing the antigens included in the array print. A template file can be downloaded from the app.
  d. Upload a print design giving the arrangement of the antigens and dilutions on the array. This file is available whilst creating the print plan in the “Print Plan Generator” section of the application.
  e. Upload the Slide Quantification for all gains associated with the slide prepared above either in Mapix of Genepix format.
    ***Note:*** The processing is designed to work with scans across multiple gains fitting a response to those gains below saturation and normalising to a calculated gain value of 50. If only 1 gain is uploaded the intensity for that gain only will be reported.
  f. Provide a slide name to identify the dataset being uploaded.
  g. Provide a sample plate name for reference to the serum samples assessed on the slide.
  h. Select the Immunoglobulin used as the detection antigen i.e., IgG.
  i. Select the intensity metric used for the analysis of the data. This is a column from the uploaded scan data. In the data presented here, the median fluorescence intensity at 785 corrected by background intensity at 785 “f785_median_b785” was used.
  j. Click on “Process Import Data”. Once the data has been processed and fitted, an overview plot of the imported data will display in the “Import Data” box.

3. Append multiple antigen microarray slides together.
  a. By clicking “Append” the uploaded antigen microarray slide data will be joined to the full dataset.
  b. In the “Full Dataset” box a list of the appended slides is reported along with buttons to download the dataset and clear the uploaded datasets.

4. Upload Existing Data. Upload a dataset previously downloaded.
  a. Select “Previously Fitted Data Upload” to open the menu.
  b. Upload a previously downloaded current dataset.
  c. Click on “Process Import Data”. Once data has been processed an overview plot of the imported data will display in the “Import Data” box.
    ***Note:*** Depending on size the graphic generation can take several minutes.
  d. Once processed, the uploaded existing data can be joined to the full dataset by clicking on “Append import data to current dataset”.
  e. In the “Full Dataset” box a list of the appended slides is reported along with buttons to download the dataset and clear the uploaded datasets.

**Table 1:** List of data files involved in the processing and analysis of the antigen microarrays

Data	Description	Example
Print Plan	The ∗.txt file used in the array printing describing the layout of the antigen microarray	Data S3
Print design	.xlsx of the Print plan as well as dilution sample dilution information	Data S6
Antigen Names	Description of the antigens printed in the microarray	Data S7
Slide Quantification	Output of the quantification of spot intensities from Mapix software.	Data S8
Processed Output	Tabulated output of fitted values for a collection of arrays.	Data S9

## Limitations

One of the main limitations to the described protocol is the availability and quality of antigens. This is particularly relevant for emergent variants where there can be a considerable delay between their description and commercial availability. During the protocol development, we also noted higher background binding to antigens produced in non-mammalian cells. As with other techniques, our approach relies on comparable recognition of recombinantly produced antigens that are bound to a surface. While a good approximation, it may not fully represent the antigens confirmation in vivo. The methodology presented here is designed to quantify antibody recognition and cannot detect the neutralizing activity of an antibody.

## Troubleshooting

### Problem 1

Antigen samples do not fully dissolve upon resuspension (before you begin step 2).

### Potential solution

If the resuspension appears cloudy consider diluting that particular antigen, or centrifuge for 10 min at (15,000 g) at room temperature in a table top centrifuge and transfer the supernatant to a new tube to collect a pure solution. Determine the protein concentration using a protein quantification kit.

### Problem 2

Condensation forming on the nitrocellulose slides during printing (step 1c).

### Potential solution

The setting of humidity and temperature is designed to optimize the analyte’s absorption onto the nitrocellulose slides. This is a setting close, but not at the dew point, where condensation forms on the glass surface of the array. If dew forms on the slide, consider if the humidity of the room has been affected such as during a thunderstorm or consider if the climate control of the room where the printer is located is stable. If the problem persists, the settings of relative humidity and temperature can be adjusted. See the printer manufacturer’s instructions for further advice.

### Problem 3

Inconsistent spotting with missing spots of deflected spots unaligned to the array grid (step 1c).

### Potential solution

Ensure the pipette tubing is free of air bubbles. Whilst in “Interactive mode” under “Functions” select “System” and “Complete Filling of Tubing and Pipettes”.

Ensure proper wetting of the piezoelectric tip. It is advisable to run one or more blank print runs onto glass slides before running an actual print run. This improves reproducibility of the piezoelectric tip.

### Problem 4

Trapped bubbles or foaming in preparation of the print plate or sample plate (step 2).

### Potential solution

If air is trapped below the liquid during the preparation of the print plate or sample plate, it can be removed by centrifuging at 100g for 30 s to bring the liquid to the bottom of the well.

### Problem 5

**Figure 9:**
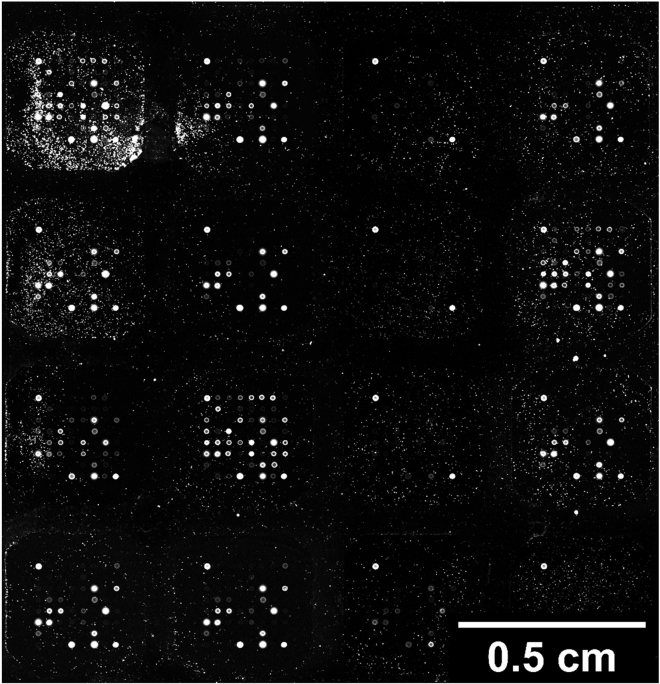
Example image of slide with poor signal to background clarity

Poor signal to noise constancy across the slide (Figure 9; step 16).

### Potential solution

It is important that the slides are not drying out during the manipulation. Work with one slide at a time to ensure rapid processing. Some residual buffer will likely be released upon separation of the ProPlate and antigen microarray slide. Use an absorbent tissue to remove the buffer. The tissue needs to be discarded according to the biosafety measures.

Be careful not to touch the microarray surface. Complete removal of the fluid is not necessary. Dispose of the fluid in a pipette basin. Ensure biological safety measures for handling primary human samples.

### Problem 6

Selection of positive pool (step 7a).

### Potential solution

Pool selection depends on the cohort in question. It is important that the pool covers all possible signals on the array at a level representative of a clear positive signal. It could be created from a subset of samples with supporting data for their positive nature for the antigens on the array or a broad selection obtaining a physical mean of the positive samples in the cohort. It should be scaled to the cohort size but should at least contain three individuals.

### Problem 7

Selection of negative pool (step 7b).

### Potential solution

A perfect negative control for a given antigen is sera from a human patient who has not been exposed to the selected antigen. This may be sera collected before the emergence of the antigen such as sera pre-2019 for SARS-CoV-2. For other antigens such a control may not be available. I such case we suggest utilization of a sera free blank.

## Resource availability

### Lead contact

Further information and requests for resources and reagents should be directed to and will be fulfilled by the technical contact, Joseph Longworth (josephlongworth@gmail.com) or the lead contact Gunnar Dittmar (gunnar.dittmar@lih.lu).

### Materials availability

This study did not generate new unique reagents.

## Data Availability

The code and example datasets generated during this study are available on at Github https://github.com/JosephLongworth/AntigenMicroarray.

## Supplemental information

Data S1. 64 pad workplate, related to step 1.a.vii

Data S2. Warm-up_print_plan, related to step 1.c.iii

Data S3. Print_plan, related to step 4.a

Data S4. InnoScan_batch_scan_method, related to step 15.b

Data S5. 64_pad_GAL, related to step 17.c.i

Data S6. Print_design, related to step quantification and statistical analysis 2.d

Data S7. Antigen_names, related to step quantification and statistical analysis 2.c

Data S8. Slide_quantifications, related to step quantification and statistical analysis 2.e

Data S9. Combinded_slide_data, related to step quantification and statistical analysis 4.e
